# Identification and validation of ecto-5' nucleotidase as an immunotherapeutic target in multiple myeloma

**DOI:** 10.1038/s41408-022-00635-3

**Published:** 2022-04-01

**Authors:** Arghya Ray, Yan Song, Ting Du, Leutz Buon, Yu-Tzu Tai, Dharminder Chauhan, Kenneth C. Anderson

**Affiliations:** The LeBow Institute for Myeloma Therapeutics and Jerome Lipper Myeloma Center, Department of Medical Oncology, Dana Farber Cancer Institute, Harvard Medical School, Boston, MA USA

**Keywords:** Cancer microenvironment, Myeloma, Myeloma

## Abstract

Interaction of plasmacytoid dendritic cells (pDCs) with multiple myeloma (MM) cells, T- or NK-effector cells in the bone marrow (BM) microenvironment induces tumor cell growth, as well as inhibits innate and adaptive immune responses. Defining pDC-MM interaction-triggered immunosuppressive mechanism(s) will enable design of interventional therapies to augment anti-MM immunity. In the present study, we show that pDC-MM interactions induce metabolic enzyme Ecto-5' Nucleotidase/CD73 in both pDCs and MM cells. Gene expression database from MM patients showed that *CD73* levels inversely correlate with overall survival. Using our pDC-MM coculture models, we found that blockade of CD73 with anti-CD73 Abs: decreases adenosine levels; activates MM patient pDCs; triggers cytotoxic T lymphocytes (CTL) activity against autologous patient MM cells. Combination of anti-CD73 Abs and an immune-stimulating agent TLR-7 agonist enhances autologous MM-specific CD8^**+**^ CTL activity. Taken together, our preclinical data suggest that the therapeutic targeting of CD73, alone or in combination with TLR-7 agonist, represents a promising novel strategy to restore host anti-MM immunity.

## Introduction

Multiple Myeloma (MM) is characterized by clonal expansion of malignant plasma cells in the bone marrow (BM) [1]. The American Society of Cancer estimated a total of 34,920 new cases of MM in the USA in 2021 [2]. The current standard of care in the treatment of MM patients include proteasome inhibitors bortezomib, carfilzomib, and ixazomib; immunomodulatory agents thalidomide, lenalidomide, and pomalidomide; histone deacetylase inhibitors panobinostat; and monoclonal antibodies daratumumab and elotuzumab [3]. While these therapies have markedly extended the survival in patients with MM, novel adjuvant immune-based therapies will achieve an efficacious and a durable anti-tumor immune response to prevent the relapse of MM [3, 4]. The interactions of MM cells with non-malignant cellular components in the host-MM bone marrow (BM) milieu such as BM accessory cells or immune cells, modulate immune-checkpoint and metabolic pathways to both reduce anti-tumor immunity and promote tumorigenesis [5–16]. Recent studies highlighted a role of one such metabolic pathway that mediates generation of immunosuppressive adenosine in the tumor microenvironment [17–20].

Adenosine is generated upon conversion of nucleotides adenosine triphosphate (ATP) and nicotinamide dinucleotide (NAD+) *via* catabolic activity of metabolizing enzymes ectonucleotidases. Specifically, CD39/ectonucleoside triphosphate diphosphohyrolase-1 converts ATP/ADP to adenosine monophosphate (AMP), followed by conversion of AMP to adenosine by CD73/ecto-5' nucleotidase/*NT5E*. Similarly, NAD+ to adenosine conversion occurs *via* sequential activity of CD38 (NAD glycohydrolase) and CD73. Prior studies showed that ATP-NAD^**+**^/ectonucleotidases/adenosine-signaling axis confer immune suppression in cancers, especially in hypoxic tumor microenvironment [21, 22]. Indeed, elevated levels of adenosine have been observed in various cancers, including MM [23–25]. Our previous studies showed that cell–cell contact between immunologically defective plasmacytoid dendritic cells (pDCs) with MM cells, T- or NK-effector cells in the BM milieu induces MM cell proliferation, as well as inhibits innate and adaptive immune responses [5, 7]. Our preclinical studies have also identified several molecular mechanism(s) including immune checkpoints mediating pDC-MM interactions; conversely, we have validated checkpoint inhibition to restore pDC immune function and inhibit MM cell growth [5–10]. In concert with our studies, a recent study using Vk*MYC myeloma mouse model showed a role of pDCs in MM progression [26]. To date, however, the role of adenosine signaling during pDC-T-MM cell interactions in MM BM microenvironment, remains undefined.

In the present study, we utilized our preclinical co-cultures models of BM accessory cells such as pDCs, or T cells, with autologous patient MM cells to examine: (1) whether pDC-T-MM cells interactions involve adenosine signaling; and (2) whether pharmacological blockade of adenosine-signaling pathway alters the anti-MM immunity. For our studies, we analyzed genetic alteration in MM cells after coculture with pDCs using RNAseq-based gene expression profiling (GEP) studies. pDC-MM interactions induce transcription of CD73/ecto-5' nucleotidase/*NT5E* in the immunosuppressive adenosine pathway. To assess the functional significance of these findings, we used our pDC/MM, or pDC/T, coculture models, and show that targeting CD73 generates MM-specific cytotoxic T lymphocyte (CTL) activity against MM cells. Moreover, the combination of anti-CD73 antibodies (Abs) with an immune activator TLR-7 agonist enhances anti-tumor immunity and cytotoxicity in MM. Our data, therefore, provide evidence for a role of CD73/ecto-5’ nucleotidase in immune modulation and provide the preclinical rationale for therapeutic targeting of CD73-mediated adenosine pathway to enhance tumor cytotoxicity and improve patient outcome in MM.

## Materials and methods

### Purification of MM patient BM pDCs, T cells, and CD138^+^ tumor cells

Studies involving patient MM cells were performed following IRB-approved protocols at Dana-Farber Cancer Institute and Brigham and Women’s Hospital (Boston, MA, USA). Informed consent was obtained from all patients in accordance with Helsinki protocol, and patient samples were de-identified prior to their use in experiments. pDCs were isolated from BM by magnetically activated cell sorting using CD304 (BDCA-4/Neuropilin-1) microbeads kit (Miltenyi Biotec, Auburn, CA, USA), as previously described [5, 7, 9]. The purity of pDCs (CD3−, CD14−, CD19−, CD20−, CD56−, CD11c−, MHC-II/CD123/BDCA-2^**+**^) was confirmed, as previously described [5, 7, 9]. Raw data was analyzed using FACS Diva (BD Biosciences) and FlowJo (Tree Star Inc, USA). MM patient cells were purified (>95% purity) by positive selection using CD138 Microbeads kit. CD8^**+**^ T cells and cells were purified using negative selection immunomagnetic separation techniques, as previously described [6–10, 12].

### Cell culture and reagents

MM-pDCs were co-cultured either in DCP-MM medium (Mattek Corp. Ashland, MA) or complete RPMI-1640 medium supplemented with IL-3 (Peprotech Inc., Rocky Hill, NJ, USA). CD3-PE/FITC/APC; CD4-FITC/PE or APC-Cy7; CD8-APC/FITC, CD56-PE; CD123-PE/PE-Cy5/FITC; and CD138-FITC/PE/APC were obtained from BD Biosciences (San Jose, CA). BDCA-2-FITC and CD11c-APC were obtained from Miltenyi Biotec (Auburn, CA); CD303-, CD304-, CD107a, CD138-BV421, and PD-L1-BV421 were purchased from Biolegend. Immunomagnetic separation kits were purchased from Miltenyi Biotec. The CellTrace Violet and CellTracker Violet/Green flow assay kits were obtained from Life Technologies (USA). CD73 blocking antibody (anti-human CD73 Abs) and TLR-7 agonist were obtained from eBiosciences and Invivogen, respectively. WST-1 Cell Proliferation Reagent was purchased from Clontech Laboratories, Inc. (USA).

### RNA sequencing using next-generation sequencing

Purified MM patient pDCs were co-cultured with autologous MM cells or allogeneic MM cell lines (1pDC:5MM) for 48 h, followed by separation of MM cells from pDCs using flow cytometry. Total RNA from MM cells was subjected to RNAseq analysis using Illumina next-generation sequencing (NGS). Raw sequence data were analyzed using VIPER [27] workflow generating differential expression (DEseq2) and KEGG pathway. The Linear model for RNAseq analysis (Limma) and its GUI (Glimma) were used for the visualization of data. Statistical significance: log2FC (fold change) values in coculture vs control, with an FDR false discovery rate value of <0.05, was considered significant (CI > 95). Pathway analysis was done using PATHVIEW (https://pathview.uncc.edu/) or pathview R package [28]. The heat map analysis was also performed using Morpheus software (Broad Institute, MIT). Gene expression studies were validated at the protein levels using multicolor flow cytometry.

### Gene expression analysis

The gene expression analysis was performed using publicly available MM patient Gene databases. The data available in the CanEvolve website is based on TT2 patients’ survival/gene expression data collected using [HG-U133_Plus_2] Affymetrix Human Genome U133 Plus 2.0 Array platform (http://www.canevolve.org/AnalysisResults/AnalysisResults.html).

We also utilized RNAseq expression database on CD138^**+**^ MM available on MMRF CoMMpass (version IA13) patients’ survival data platform (MMRF Research Gateway website: https://research.themmrf.org/; MMRF CoMMpass data version IA13).

### Adenosine assays

MM patient total BM-MNCs or CD138^**+**^ MM cells were treated with anti-CD73 Ab (0.5 µg/ml) or isotype control Abs for 48 h; supernatants were subjected to Adenosine assay using human Adenosine assay kit (Abcam Inc, MA, USA), as per manufacturer’s protocol. Data presented shows change in adenosine conversion in supernatants from BM-MNCs or CD138^**+**^ MM cells treated with anti-CD73 Abs *versus* isotype control Abs. Adenosine measurement in coculture assays: MM BM pDCs were co-cultured with CD138^**+**^ MM cells in the presence of anti-CD73 Ab (0.5 µg/ml) or isotype control for 48 h; supernatants were subjected to Adenosine assay. Data presented shows change in adenosine conversion in supernatants from pDCs-MM co-cultures treated with anti-CD73 Abs *versus* isotype control Abs.

### Flow cytometry analysis and cell viability assays

MM cells were co-cultured for 24 h with pDCs; cells were then stained with CD73-FITC and CD138 Abs, followed by multicolor flow analysis to quantify CD73^**hi**^ MM cell populations. Cell viability was assessed by WST assay, as previously described [5, 7, 9, 11].

### Cytotoxic T Lymphocyte (CTL) activity assays

MM patient BM CD8 + T cells were co-cultured for 3 days with autologous pDCs at 1:10 (pDC:T) ratio, in the presence or absence of anti-CD73 Ab (0.5 µg/ml) or TLR-7 agonist (0.1 µM). After washing to remove drugs, cells were cultured for 24 h with autologous MM cells pre-stained with CellTrace Violet or CellTracker Violet (E/T ratio: 10:1, T: MM), followed by 7-AAD staining and quantification of MM cell lysis by FACS, as previously described [5, 7, 9].

### Statistical analysis

Statistical significance was obtained using Student’s *t*-test, with the minimal level of significance at *p*-value < 0.05 (Graph Pad PRISM version 8).

## Results and discussion

### pDCs trigger transcriptomic alterations in MM cells

**Fig. 1:**
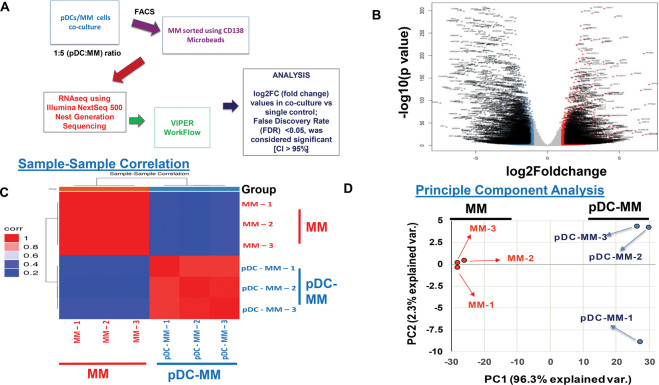
RNAseq analysis of MM cells in the presence or absence of pDCs. **A** Schema showing an overview of RNAseq methods and bioinformatics analysis. Purified MM patient pDCs (from three MM patients) were co-cultured with MM.1S cells (1pDC:5MM) for 48 h, followed by separation of MM.1S cells from pDCs using flow cytometry. Total RNA from MM.1S cells was subjected to RNAseq analysis using Illumina NGS. Raw sequence data were analyzed using VIPER workflow generating differential expression (DEseq2) and KEGG pathway [27, 28]. Statistical significance: log2FC (fold change) values in coculture vs control, with an FDR false discovery rate value of <0.05, was considered significant (CI > 95). Linear model for RNAseq analysis (Limma) and its GUI (Glimma) were also utilized for the visualization of data. **B–D** pDCs trigger genetic alterations in MM cells. Quantitative analysis demonstrated upregulation or downregulation of genes in MM cells after coculture with pDCs (log2FC range: ±6.0). MM.1S cells were co-cultured with pDCs (from three MM patients) for 48 h (in triplicate, *n* = 3); separated using CD138 antibody by flow sorting, and harvested. Poly RNA was subjected to RNAseq analysis using Illumina NextSeq 500 NGS for total RNA. A total of 19,569 and 25,436 transcripts were analyzed through negative binomial (DEseq2) and linear (Limma) models, respectively. We found that ~9200 (47.01%) and 9250 (36.36%) genes, respectively, were differentially upregulated or downregulated (*p* < 0.05). **B** Volcano Scatter plot showing RNAseq data for MM.1S alone versus coculture. The expression given in log_2_FC varies from ±6 log fold change in MM.1S cells after coculture. An FDR false discovery rate adjusted *p*-value < 0.05 is considered significant. Data presented are an average of three samples in each group. Significant gene expression changes are at the top of the plot. Genes are colored red if the log2 fold change is >1 (log2FC > 1). Genes are colored blue if the log2 fold change is <−1 (log2FC < −1). **C** Sample–Sample correlation for MM.1S cells cultured in the presence (pDC-MM or coculture; sample names: pDC-MM-1, pDC-MM-2, and pDC-MM-3) vs absence (MM; sample names: MM-1, MM-2, and MM-3) of pDCs is shown. The correlation clustering shows that MM and pDC-MM are clustered in two different groups based upon differential gene expression. **D** Principal component analysis (PCA) showing pDC-induced genetic changes in MM cells: high dimensional expression data was mathematically reduced to principal components that were used to describe variation across samples in fewer dimensions to allow logical interpretation. Principal component 1 (PC1) accounts for the most amount variation across samples, followed by PC2. The PC1 vs PC2 plot of the RNAseq data is presented showing two sets of samples (MM and pDC-MM) clustered in spatially separated groups in reduced dimensional space, implicating a distinct genetic status of MM cells when cultured alone (MM) versus with pDCs (pDC-MM).

Purified MM patient pDCs (*n* = 3; three different relapsed/refractory multiple myeloma patients) were first co-cultured with MM.1S cells (1pDC:5MM) for 48 h, followed by separation of MM.1S cells from pDCs using flow cytometry. Total RNA from MM.1S cells was subjected to RNA sequencing (RNAseq) analysis using Illumina NGS. As shown in Fig. 1A, raw sequencing data were analyzed with bioinformatics program VIPER (Visualization Pipeline for RNAseq analysis) [27]. We analyzed differentially expressed genes in MM cells cultured in the presence versus absence of pDCs. For RNAseq analysis, log2FC (fold change) values in coculture vs single control with an FDR false discovery rate adjusted *p*-value of <0.05 was considered significant (CI > 95). We used negative binomial distribution (DEseq2) and linear (Limma) statistical models for our RNAseq analysis. A total of 19,569 and 25,436 transcripts were analyzed through negative binomial (Deseq2) and linear (Limma) models, respectively; 9200 (~47.01%) and 9250 (~36.36%) genes, respectively, are differentially expressed with an adjusted *p*-value < 0.05. Since the adjusted *p*-values are <0.05, the alternations in these genes were considered significant irrespective of their log2FC (fold change) values (Supplemental data file 1).

Further examination showed that MM cells co-cultured with pDCs exhibit a distinct transcriptional profile versus MM cells cultured alone, evidenced in three distinct analyses: (1) A volcano plot analysis to simultaneously assess the statistical significance (*P*-values) and biological difference (log ratios) of differentially expressed genes showed a large number of genes to be highly upregulated (Log2FC > 1; red) or downregulated (Log2FC < −1; blue) in MM cells cultured with pDCs versus without pDCs (Fig. 1B and Supplemental data file 2); (2) Sample correlation coefficient analysis (heat map) based on pairwise basis of all samples showed that MM cells cultured with or without pDCs fall into two genetically distinct clusters (Fig. 1C); and (3) A predictive model based on principal component analysis (PCA) of RNAseq data showed that MM cells cultured with or without pDCs segregate into two distinct spatially separated principle axes/groups (PC1 and PC2) (Fig. 1D). Together, these analyses demonstrate that pDCs induce transcriptional changes in MM cells.

**Fig. 2:**
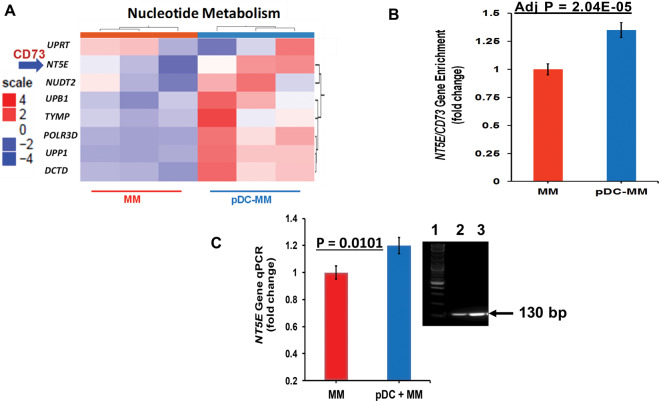
Effects of pDC-MM interaction on nucleotide degradation pathway-related genes and clinical relevance of CD73 in MM. **A** Purified MM patient pDCs (from three MM patients) were co-cultured with MM.1S cells (1pDC/5MM) for 48 h, followed by separation of MM.1S cells from pDCs using flow cytometry. Total RNA from MM.1S cells was subjected to RNAseq analysis as in Fig. 1A. Hierarchical clustering analysis of RNAseq data show that pDCs alter purine/pyrimidine nucleotide degradation-related genes including ectoenzyme *NT5E/CD73* in MM cells. **B** *CD73* gene enrichment in pDC-MM cell coculture versus MM cells alone [1.34-fold upregulation; *n* = 3; Adjusted *p* = 2.04E -05]. **C** Validation and Quantification of NT5E gene expression by RT-qPCR: MM cells were co-cultured with pDCs for 48 h (in triplicate, *n* = 3); separated using CD138 antibody by flow sorting, and harvested. Poly RNA was subjected to RT-qPCR using Luna Universal 1-step RT-qPCR kit (New England BioLabs, MA, USA) following manufacturer protocol using an Applied Bioscience 7500 Fast Real-Time PCR System (Thermo Scientific Inc). *NT5E/CD73* gene expression was quantified from the raw data using ΔΔCT and utilizing GAPDH as the housekeeping reference gene control under the experimental conditions. The bar graph denotes NT5E. gene expression in MM cells cultured in the presence vs absence of pDCs (mean ± SD; *p* < 0.05). Inset: Analysis of qPCR amplicons (130 bp) on a 2.5% agarose gel stained with GelRed Nucleic Acid Gel Stain (Biotium Inc, USA). Lane-1: 1 kb DNA ladder; Lane-2 and lane-3: NT5E expression in MM cultured in the absence and presence of pDCs, respectively.

Gene Ontology and KEGG pathway analysis of RNAseq data provided evidence for the functional relevance of pDC-triggered genetic changes in MM cells (Supplementary Fig. 1 and Fig. 2). For example, our previous studies showed that pDCs induce growth, survival, drug-resistance, and immune suppression in MM [5–10, 12]; and importantly our current RNAseq analysis found that pDCs trigger changes in genes promoting such biologic signaling pathways in MM cells (e.g., MAPK1, MDM2, WDR48, MLH1, BCL2, BAK1, TOP2A, PD-L1, TLR-7/9, IL-3-Rα/CD123, ENO1, or HDAC6). Of note, our prior studies identified and validated some of these molecules (e.g., PD-L1, TLR9, HDAC6, or ENO1) as therapeutic targets to restore anti-MM immunity [5–10, 12].

### pDC-MM interactions induce transcriptional changes in adenosine-signaling pathway enzyme ecto-5' nucleotidase (CD73) with clinical translational relevance

Recent reports highlighted a role of ectonucleotidases in energy metabolism pathways in hematologic malignancies, especially in the context of the tumor microenvironment [20, 29–32]. We, therefore, next analyzed our RNAseq data to determine whether pDCs modulate energy metabolic ectonucleotidase pathway molecules in MM cells to confer tumor growth and immune suppression. Expression patterns for MM cells cultured with or without pDCs were compared, and a heat map was generated (CI > 95%). As shown in Fig. 2A and Supplementary Fig. 2, pDCs alter transcription of adenosine-linked metabolic/purinergic pathway genes in MM cells. The Supplementary Fig. 2A shows all the major pathways that are primarily affected in MM after coculture with pDCs, including nucleotide metabolism pathway. The heat map indicated both upregulated and downregulated genes. Figure 2 shows a partial heat map for the genes including *NT5E/CD73* that are upregulated in MM after coculture with pDCs (Supplementary Fig. 2B).

Importantly, pDCs increase *NT5E/CD73*, a nucleotidase required for catalysis of both ATP and NAD^**+**^ nucleotides into adenosine, in MM cells (Fig. 2B, Bar graph; 1.34-fold increase in pDCS with MM cells versus MM cells alone; *n* = 3; adj *p* = 2.04E-05). pDC-induced *CD73* transcription in MM cells was confirmed using RT-PCR analysis (Fig. 2C; *p* = 0.0101; *n* = 3). Although our data show changes in other purinergic signaling-associated genes, we focused on delineating the role of CD73 in the present study since: (1) CD73 is a common downstream ectonucleotidase mediating adenosine generation via both ATP- and NAD^**+**^-signaling axis triggered by CD39 and CD38, respectively; (2) adenosine is elevated in the MM BM milieu, which may contribute to immune deficiency characteristic of MM; (3) CD73 is expressed on exosomes [33], which in turn promotes adenosine generation and activation of adenosine receptors on immune cells, thereby mediating immune suppression; (4) CD73 is essential for maintaining tumor cell metabolism *via* regulating adenosine homeostasis, and represents a potential immunotherapeutic target in cancer; (5) Studies in solid tumor models have linked CD73 to tumorigenesis and drug-resistance [20, 30]; conversely, genetic deletion of CD73 increases anti-tumor immune responses [30]; (6) CD73 inhibition promotes dendritic cell-mediated anti-tumor activity [29]; and finally, (7) Preclinical evaluation of the biologic function and underlying molecular mechanisms of CD73 in MM is feasible due to availability of agents targeting CD73 [34].

Consistent with our findings in MM, high *CD73* expression has been linked with poor prognosis in patients with breast, lung, pancreas, gastric, or colon cancer [35–39]. These observations suggest the potential use of CD73 as a biomarker for CD73-targeted therapies. Importantly, our findings showing increased CD73 expression due to pDC-MM interactions indicate enhanced adenosine-signaling in the MM BM milieu. It is known that CD73 can be transcriptionally regulated by (transcription) factors that are capable of binding to the DNA regulatory sequences in NT5E promotor region [40]. Both HIF1a and PCBP2 are downregulated in MM after coculture with myeloma pDC (Supplemental Data File 1). Several members of the SMAD family of transcription factors are known to be directly associated with the regulation of CD73 expression [40]. Our RNAseq results show that the transcriptional activators SMAD4/SMAD3/SMAD2 are upregulated in MM tumors after coculture with pDCs. Another transcription factor SP1, which is known to activate *NT5E/CD73* [40] is also upregulated in MM after pDC coculture (Supplemental Data File 1). It is also known that *NT5E/CD73* expression can be regulated by ETS family of transcription factors. Our analysis further suggests the gene expression of several ETS family proteins is altered in MM after coculture with pDCs. (Supplemental Data File 1). Taken together we can conclude that all these transcriptional factors can contribute directly or indirectly to the regulation of CD73 expression in MM after coculture with pDCs.

### Validation and functional significance of CD73 in MM

**Fig. 3:**
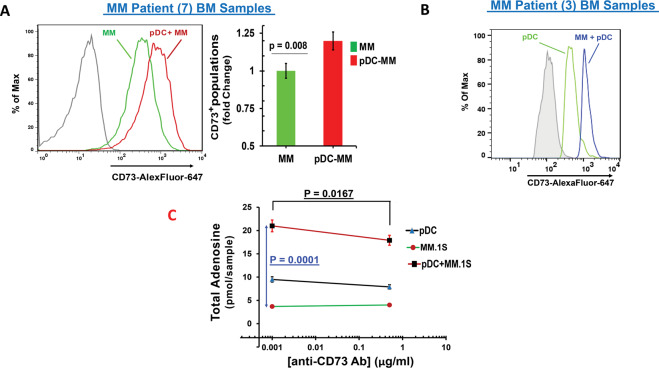
Modulation of CD73 expression and adenosine levels during pDC-MM cell interactions. **A** pDCs were co-cultured with autologous patient MM cells for 24 h, followed by multicolor flow analyses to assess the pDC-induced change in CD73 expression on MM cells. CD138^**+**^ MM cells were examined using flow cytometry, and Median Fluorescence Intensity (MFI) of CD73 expression was determined using anti-CD73 Abs conjugated to AlexaFluor-488, in the presence or absence of pDCs. *Left Panel:* Representative histograms show CD73 expression in MM cells cultured in the presence (red) and absence (green) of pDCs. [Black histogram: Isotype control Ab]. *Right panel:* Normalized CD73 expression in MM cells cultured in the presence and absence of pDCs. Data were quantified from the histogram analyses shown in the left panel (mean ± SD; *p* = 0.008; MFI: 1.2-fold change in pDC-MM vs MM; Data obtained from analysis of seven MM patient BM samples). **B** pDCs were co-cultured with autologous patient MM cells for 24 h, followed by multicolor flow analyses to assess the changes in CD73 expression on CD303 + MM BM pDCs. Median Fluorescence Intensity (MFI) of CD73 expression was determined using anti-CD73 Abs conjugated to AlexaFluor-647. Representative histograms show CD73 expression in MM BM pDCs cultured in the presence (blue) or absence (green) of MM cells. (Shaded histogram: Isotype control Abs). **C** MM- BM pDCs were co-cultured with MM cells in the presence and absence of anti-CD73 Abs (0.5 µg/ml) for 2 days; supernatants were collected and analyzed for adenosine levels using a fluorometric adenosine assay kit. The percentage of soluble adenosine in isotype-Ab- versus anti-CD73 Ab-treated MM cells, MM BM pDCs, both alone, or in pDC-MM coculture (mean ± SD, *p* < 0.05; *n* = 3) is shown. The quantification of data showing the amount of adenosine in supernatants (pmol/sample) for MM-pDC coculture versus MM cells alone (*p* = 0.0167). (Note: The isotype treatment is indicated as a very low level of anti-CD73 Ab in the semi-log plot).

We next assessed whether pDC-induced *CD73* gene levels in MM cells correlate with alterations in CD73 protein expression. Freshly isolated MM patient pDCs were co-cultured with autologous MM cells, followed by analysis of CD73 expression using flow cytometry. Results showed that MM cells cultured with pDCs exhibit a marked increase in CD73 expression; moreover, pDCs also increase the number of CD73^**+**^-expressing MM cells (Fig. 3A, histogram and bar graph, respectively; *p* = 0.008). Interestingly, CD73 levels were also increased in pDCs after their coculture with MM cells (Fig. 3B). Of note, pDCs from normal healthy donors also enhance CD73 expression in MM cells, albeit to a lesser extent than MM patient pDCs (data not shown). The fact that CD73 mediates conversion of ATP to adenosine, together with our data showing increased CD73 in pDC-MM co-cultures, suggests that pDC-MM interactions may modulate the generation of immunosuppressive adenosine *via* enhanced CD73.

To determine whether pDC-MM interactions enhance adenosine generation, MM.1S were next cultured with or without MM patient pDCs for 2 days, followed by measurement of adenosine levels in the supernatants using fluorometric adenosine assays. A significant increase in adenosine levels was noted in supernatants from MM-pDC co-cultures versus MM cells cultured alone (5.5-fold increase) (Fig. 3C). Conversely, blockade of CD73 using anti-CD73 Ab significantly reduced (~18%) adenosine levels in pDC-MM co-cultures in a concentration-dependent manner (Fig. 3C; *p* = 0.0167). As a control, we have also evaluated the levels of adenosine in pDC alone in the presence or absence of anti-CD73 Ab (Fig. 3C). The adenosine level in the supernatant from pDC culture is higher than that in the supernatant from MM cells cultured alone. Importantly, the blockade of CD73 using anti-CD73 Ab also reduced adenosine levels (~16%; *p* = 0.0109) in pDC culture supernatant. We also performed additional studies with MM patient-derived total bone marrow mononuclear cells (MM BMSCs) treated with anti-CD73 Ab at various concentrations. Our results show that anti-CD73 Ab triggers a dose-dependent decrease in adenosine levels in MM BMNCs (Supplementary Fig. 3). Taken together, our data suggest that pDC-MM interactions increase both CD73 expression and adenosine generation; and importantly, that CD73 inhibition partially decreases adenosine production at this concentration, even in the context of pDC/MM cell co-cultures.

### Inhibition of CD73 activates MM patient pDCs and stimulates T cell activation

**Fig. 4:**
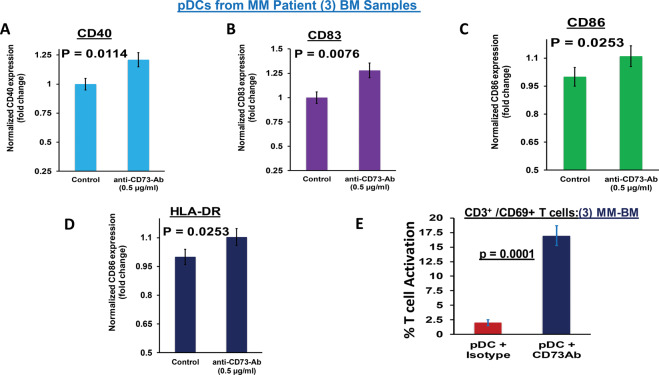
CD73 blockade activates pDCs and enhances pDC-triggered T cell proliferation. (**A–D**) MM patient pDCs (*n* = 3) were treated with anti-CD73 Ab (0.5 µg/ml) for 24 h, followed by multicolor staining. Expression of pDC activation/maturation markers CD40, CD83, CD86, and HLA-DR were assessed using flow cytometry. Fold change in treated versus untreated pDCs is shown (mean ± SD; *p* < 0.05; *n* = 3). **E** MM patient pDCs (*n* = 3) were co-cultured for 3 days with autologous T cells at 1:10 (pDC:T) ratio, in the presence or absence of anti-CD73 Ab (0.5 µg/ml). Viable CD3^+^ T cells were analyzed for the expression of CD69 activation marker using anti-CD3-FITC and anti-CD69-APC-Cy7 Abs, and quantified by FACS (mean ± SD; *P* < 0.05, *n* = 3). [Note: The percentage of CD3 + /CD69 + T cells denotes activated T cells in the presence of anti-CD73 Ab; three myeloma bone marrow (MM BM) samples were used].

A recent study showed that targeting CD73 enhances dendritic cell-mediated anti-tumor activity [29]. Since MM-pDCs exhibit reduced ability to trigger T cell proliferation [5–9], we next determined whether CD73 blockade restores pDC immune function and triggering of immune effector cells. As shown in Fig. 4A–D, treatment of MM patient pDCs with anti-CD73 Ab activates pDCs, evidenced increased expression of maturation/activation markers (CD40/CD83/CD86/HLA-DR). Importantly, treatment of MM patient pDCs with anti-CD73 Ab restores their ability to trigger the activation of autologous T cells (Fig. 4E; mean ± SD; *n* = 3, *p* < 0.05).

### Blockade of CD73 triggers pDC-mediated T cell-cytolytic activity against MM cells

**Fig. 5:**
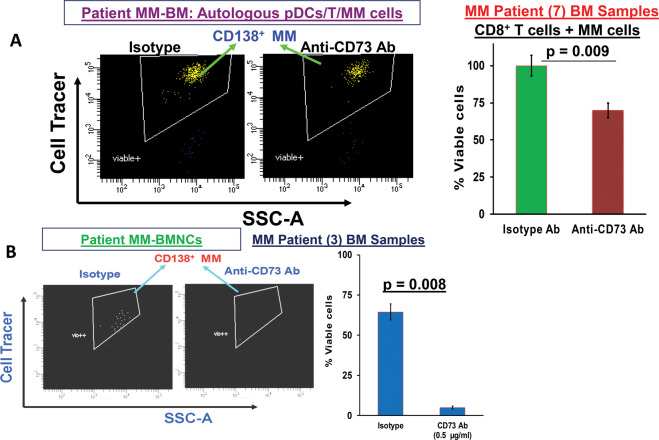
Blockade of CD73 induces T cell-mediated autologous MM cell killing. **A** MM patient (*n* = 7) BM CD8^+^ T cells were co-cultured with autologous pDCs (1pDC: 10 T cells), in the presence or absence of anti-CD73 Ab (0.5 µg/ml) for 3 days. After washing to remove anti-CD73 Ab, cells were cultured for 24 h with autologous MM (CD138^**+**^) cells pre-stained with CellTracker Violet (T/MM; 10:1 ratio), followed by 7-AAD staining, and quantification of CTL-mediated MM cell lysis by FACS. The 7-AAD + populations were gated out and viable 7-AAD negative cells were further gated to quantify 7-AAD negative CellTracker Violet+ cells. Left panel: representative FACS scatter plot showing the decrease in the number of viable 7-AAD negative/Cell Tracker-positive MM cells. Right panel: bar graph shows quantification of CD8^+^ CTL-mediated MM cell lysis, reflected in decreased CD138 + MM cell viability after anti-CD73 Ab treatment, using data obtained in left panel (mean ± SD; *p* < 0.05). **B** MM patient (*n* = 3) total BM-MNCs were treated with anti-CD73 Ab (0.5 µg/ml) or isotype-Ab for 2 days, and multicolor flow analysis was utilized to assess MM cell lysis. CD138^+^ MM cells were quantified by staining with CD138-FITC Ab. Left panel*:* representative FACS scatter plot showing a decrease in number of viable FITC-positive MM cells after anti-CD73 Ab treatment. Right panel*:* bar graph shows quantification of CD138^+^ MM cells in left panel. The fold change was obtained after normalization with control data, and presented as a percentage of viable cells in the presence versus absence of anti-CD73 Ab (mean ± SD; *p* < 0.05).

We have previously shown that activation of pDCs upon treatment with immunomodulatory agents can stimulate T cell immune function in MM [5–10, 12]. We therefore next further examined whether anti-CD73 Ab-activated pDCs can trigger cytotoxic T lymphocyte (CTL) activity. These studies were carried out using co-cultures of freshly isolated patient pDCs, T cells, and autologous MM cells. For these studies, we obtained samples from seven MM patients, including two patients with newly diagnosed untreated MM, and five patients with relapsed MM resistant to lenalidomide, pomalidomide, bortezomib, dexamethasone, and CD38 monoclonal antibody (MoAb) therapy. MM patient BM CD8 + T cells were co-cultured for 3 days with autologous pDCs (*n* = 7, 1 pDC: 10 T cells), in the presence or absence of anti-CD73 Abs (0.5 µg/ml). After washing to remove the anti-CD73 Ab, cells were cultured for 24 h with autologous MM cells pre-stained with CellTracker or CellTrace Violet (10 T: 1 MM), followed by 7-AAD staining and quantification of MM cell lysis by FACS. Targeting CD73 induced significant MM-specific CD8^+^ CTL activity, evidenced by decreased viable MM cells (Fig. 5A; scatter plot and bar graph; *p* = 0.009). Furthermore, blockade of CD73 also decreased viable MM cells in patient BM-MNCs (Fig. 5B; *n* = 3; *p* = 0.008), suggesting that targeting metabolic pathway enzyme CD73 may impact the MM BM microenvironment and thereby evoke an anti-MM immune response. Importantly, our findings show that blockade of CD73 activates pDCs and restores pDC-induced T cell-mediated cytolytic activity against MM cells. It is noteworthy to mention here that anti-CD73 Ab does not have any direct cytotoxic effect on MM cells in the range of the concentrations used for the CTL assays (Supplementary Fig. 4). Similar results were obtained for CD138-negative bone marrow stromal cells treated with various concentrations of anti-CD73 Ab (data not shown).

It is worth mentioning here that adenosine receptors are highly expressed in MM tumor microenvironment and our results show that pDCs have a high expression of A2b receptors (Supplementary Fig. 5). We hypothesized that the adenosine signaling can also be blocked in MM via targeting of the A2b receptors. Our pDC-T coculture model will be utilized in future studies to trigger MM immune-cell death by targeting A2bR and thereby blocking immunosuppression via adenosine signaling.

### Combination of anti-CD73 Ab and Toll-like receptor-7 agonist increases MM-specific CTL activity

**Fig. 6:**
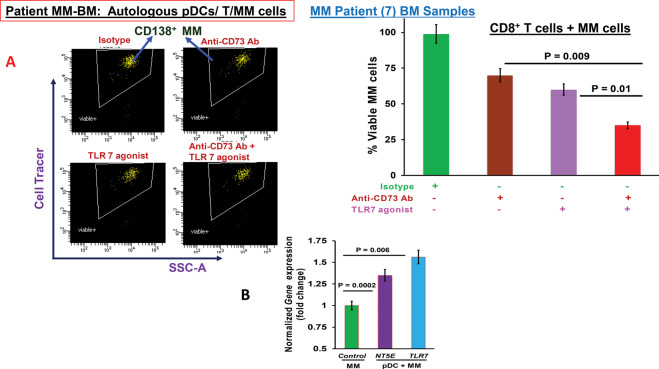
Combination of anti-CD73 Ab and TLR-7 Agonist enhances T cell-mediated MM-specific cytotoxic activity. **A** MM patient (*n* = 7) BM C8^**+**^ T cells were co-cultured for 3–5 days with autologous pDCs (1 pDC: 10 T cells) in the presence of isotype control Ab, anti-CD73 Ab, TLR-7 agonist, or anti-CD73 Ab plus TLR-7 agonist. Cells were washed and resuspended in a complete medium without drugs, and then cultured with autologous MM cells (10 T: 1 MM cell) pre-stained with CellTrace violet for 24 h, followed by 7-AAD staining and FACS analysis to quantify CTL-mediated MM cell lysis. Left panel: representative FACS scatter plot shows a decrease in number of viable CellTrace Violet-positive MM cells. MM cells were also cultured alone without immune effector cells for 24 h, and viability data obtained from flow analysis was used for normalization and account for spontaneous MM cells death. Right panel: bar graph shows quantification of CD8^+^ CTL-mediated MM cell lysis, reflected in % Viable MM cells under different treatment conditions, using data obtained in left panel. Percentage of viable MM cells for each treatment versus control (isotype-Ab) is presented. **B** Bar graph shows normalized *NT5E/CD73 and TLR-7* gene enrichment in pDC-MM cell co-cultures versus MM cells alone.

The immunosuppressive BM microenvironment contributes to the induction of tolerogenic pDCs in MM [3, 5–16]. We have previously shown that TLR-7/9 ligation stimulates pDC maturation and restores their immune function [5, 6]. Specifically, our prior studies showed that treatment of MM-pDCs with TLR9 agonist activates pDCs, which in turn increases T cell proliferation and CTL activity against MM cells [5, 6]. Based on these prior observations and our present findings, we hypothesized that combined immune stimulation via TLR ligation and blockade of immunosuppressive pathway via CD73 may enhance an anti-MM immune response. For these combination studies with anti-CD-73 Ab, we utilized a proof-of-concept small-molecule TLR-7 agonists [41, 42]. MM patient BM CD8^**+**^ T cells (*n* = 7) were co-cultured for 3 days with autologous pDCs (1 pDC:10 T cells) in the presence of isotype control Ab, anti-CD73 Ab, TLR-7 agonist, or anti-CD73 Abs plusTLR-7 agonist, and then evaluated in CTL assays against autologous MM cells (10 T: 1 MM cell). The combination of anti-CD73 Ab and TLR-7 agonist triggered more robust autologous MM-specific CD8 + CTL activity than either agent alone (Fig. 6A; scatter plot and bar graph). Of note, we found a similar extent of TLR-7 gene enrichment as for CD73 (Fig. 1) in pDC-MM cell co-cultures versus MM cells alone (Fig. 6B). These combination studies of anti-CD73 Ab with TLR-7 agonist, therefore, suggest the potential clinical utility of this combination to enhance anti-MM immunity. Importantly, our pDC-T coculture model can also be used to trigger immune cells-mediated MM cell death by targeting A2bR in combination with TLR-7 agonists to block immunosuppression (Supplementary Fig. 6; *n* = 2; *p* < 0.05 for combo *versus* single agent).

**Fig. 7:**
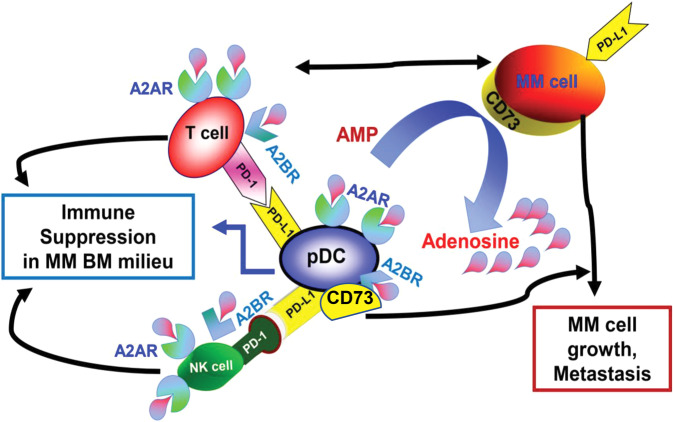
Schematic representation of the immunosuppressive adenosine-signaling pathway in MM bone marrow microenvironment. Schema shows modulation of immunosuppressive adenosine-signaling pathway via CD73 during pDC-T-NK-MM cells interactions, as well as therapeutic utility of targeting CD73 in MM.

Collectively, we here show that (1) pDC-MM interactions induce transcription of adenosine-signaling pathway enzyme ecto-5' nucleotidase CD73 in MM cells (Fig. 7, schema); (2) both pDCs and MM cells express CD73, and pDC-MM interactions further increase CD73 levels in both cells; (3) pDC-MM interactions increase adenosine generation; and importantly, CD73 inhibition decreases adenosine production; (4) CD73 blockade activates MM patient pDCs and triggers autologous T cell proliferation; (5) Targeting CD73 induces MM-specific CD8^**+**^ CTL activity; and (6) the combination of anti-CD73 Ab and TLR-7 agonist induces more potent MM-specific CD8^**+**^ CTL activity against autologous tumor cells than either agent alone. Our preclinical data, therefore, provide evidence for an immunomodulatory role of CD73 enzyme in MM, and suggests that therapeutic targeting of CD73-mediated metabolic pathways (Fig. 7, schema), either alone or in combination with TLR-7 agonist, will increase immune-mediated cytotoxicity in MM. A clinical trial of ORIC-533 oral CD73 inhibitor will soon begin in patients with relapsed refractory MM.

## Supplementary information

Supplemental-final-with Figures

Supplementary-file-1-NGS-RAW-data.csv

Supplementary-file-2-volcano-plot.pdf

Authors-agreement-on-Change-of-authorship

Authors-agreement-on-Change-of-authorship: Doc file
