# Supplementary material for: Identification and validation of ecto-5' nucleotidase as an immunotherapeutic target in multiple myeloma
Source: Blood Cancer J. 2022 Apr 1;12(4):50. doi: 10.1038/s41408-022-00635-3 (PMC8976016; doi:10.1038/s41408-022-00635-3)
Supplement: Supplementary file 1 — Supplemental-final-with Figures [file 41408_2022_635_MOESM1_ESM.pdf]

Supplemental Section:

Identification and Validation of Ecto-5' Nucleotidase as an Immunotherapeutic Target in Multiple Myeloma

[1]Arghya Ray[*], Ph.D, [1]Yan Song, PhD, [1]Ting Du, Ph.D, [1]Leutz Buon, [1]Yu-Tzu Tai, PhD, [1]Dharminder Chauhan*¶, Ph.D and [1]Kenneth C Anderson*¶, M.D.

[1]The LeBow Institute for Myeloma Therapeutics and Jerome Lipper Myeloma Center, Department of Medical Oncology, Dana Farber Cancer Institute, Harvard Medical School, Boston, MA

Running Title: Targeting Ecto-5' Nucleotidase as immunotherapy in myeloma

¶*Joint Senior authors*

Conflicts of Interest disclosure. K.C.A. is an advisor for Janssen, Amgen, Pfizer, Precision Biosciences, Mana, and Raqia; and is a Scientific Founder of OncoPep and C4 Therapeutics. DC is consultant to Stemline Therapeutic, Inc., and Oncopeptides AB, and Equity owner in C4 Therapeutics. Other authors have no competing financial interests.

* **Correspondence** Dharminder Chauhan, Ph.D (Dharminder_Chauhan@dfci.harvard.edu); Kenneth C Anderson, M.D. Kenneth_Anderson@dfci.harvard.edu); ~~Arghya Ray, Ph.D (arghya_ray@dfci.harvard.edu);~~ Dana-Farber Cancer Institute, M561, 450 Brookline Ave, Boston, MA;

Keywords: Myeloma, CD73, Immunotherapy, Plasmacytoid Dendritic Cells, Toll-like receptor

Word count: Abstract: **162**; Text: 3820; Number of Figures: **7**; Number of eferences: **42**

Supplemental Figure Legends

Supplementary Figure 1: Gene Ontology (GO) Term Analysis of different Immune Pathways MM cells. Different Immune pathways affected in MM cells cultured in the presence (**pDC-MM or coculture**) vs absence (**MM**) of pDCs is shown. The plot indicated different downregulated immune pathways in GO Term based vs the Log of their adjusted P Values (Q – values).

Supplementary Figure 2A: Combined Heatmap of five major pathways that are affected in MM after coculture with pDCs. Heatmap for each pathway was generated based on KEGG pathway analysis using Pathview software. [Ref: *Luo, Weijun, Brouwer, Cory (2013). "Pathview: an R/Bioconductor package for pathway-based data integration and visualization." Bioinformatics, 29(14), 1830-1831. doi: 10.1093/bioinformatics/btt285*.]

Supplementary Figure 2B: pDC-MM interaction Affects Nucleotide Metabolism Pathway and therein Upregulates *NT5E/CD73* in the MM cells: Panel shows the hierarchical clustering of RNAseq data corresponding to the genes in the Nucloeotide metabolism pathway including the upregulated metabolic ectoenzyme NT5E/CD73 in MM after coculture with MM.

Supplementary Figure 3 Total bone marrow mononuclear cells isolated from the MM patient bone marrow (MM-BM ; n =2) were treated with various

concentrations of anti-CD73 Abs (0.5-1.5 μg/ml) for 2 days; the isotype-Ab was used as control. Supernatants were collected and analyzed for adenosine levels using fluorometric adenosine assay kit. The fold change in soluble adenosine in isotype-Ab- versus anti-CD73 Ab-treated BM-MNCs (mean ± SD, $p < 0.05$; $n = 3$) is shown.

Supplementary Figure 4: MM cells (MM.1S and U266) were treated with increasing concentrations of anti-CD73 Ab for 72h followed by the assessment of viability using WST-1 assay. The bar plots indicate change in % viability of cells at different concentrations of anti-CD73 Ab. (mean ± SD, $P < 0.05$).
[Note: anti-CD73 Ab is non-toxic to MM cells within the range of 0-2.5 μg/ml]

Supplementary Figure 5: pDCs were stained with either anti-A2bR antibody or isotype antibody, both conjugated to AlexaFluor -647 and subjected to flow analysis to assess the A2bR expression on pDCs. *Left Panel:* Representative scatter plots show the A2bR expression on pDCs. *Right panel:* Corresponding histograms drawn from the left panels to show A2bR expression on pDCs (Data obtained 3 MM patient BM samples).

Supplementary Figure 6 Combination of A2b receptor antagonist and TLR7 Agonist induces autologous MM cell killing MM patient (n=2) total BM-MNCs were treated with isotype control Ab, A2bR antagonist (1 μM BAY-

545, MedChem, USA), TLR7 agonist, or A2bR agonist plus TLR7 agonist. For 3 days, and multicolor flow analysis was utilized to assess MM cell lysis. 7AAD-positive cells were gated out and $CD138^{+}$ MM cells were quantified by gating MM cells stained with CD138-BV421 Ab. *Left panel:* Representative FACS scatter plot showing a decrease in number of viable BV-421-positive MM cells after the treatment. *Right Panel:* Bar graph shows quantification of $CD138^{+}$ MM cells in left panel. The fold change was obtained after normalization with control data, and presented as percentage of viable cells Percentage of viable MM cells for each treatment versus control (isotype Ab) is presented. (mean ± SD; $p < 0.05$).

Supplemental Data File 1: RNA-Seq analysis of MM cells in the presence or absence of pDCs Purified MM patient pDCs (from 3 MM patients) were co-cultured with MM.1S cells (1pDC:5MM) for 48h, followed by separation of MM.1S cells from pDCs using flow cytometry. Total RNA from MM.1S cells was subjected to RNAseq analysis using Illumina Next Generation Sequencing (NGS). Raw sequence data were analyzed using VIPER workflow generating differential expression (DEseq2). Statistical significance: log2FC (fold change) values in co-culture vs control, with an FDR (False Discovery Rate) value of <0.05, was considered significant (CI > 95).

Supplemental file 1: ArghyaRay-21-BCJ-0687R -CD73-manuscript-revised-Supplementary-file-1-NGS-RAW-data.csv

Supplemental Data File 2: Volcano Scatter plot showing RNAseq data for MM.1S alone versus co-culture. The expression given in log_2FC varies from ± 6 logFoldchange in MM.1S cells after coculture. An FDR (False Discovery Rate) adjusted p-value < 0.05 is considered significant. Data presented is an average of 3 samples in each group. Significant gene expression changes are at the top of the plot. Genes are colored red if the log2 fold change is greater than one (log2FC > 1). Genes are colored blue if the log2 fold change is less than negative one (log2FC < -1).

Supplemental file 2: ArghyaRay-21-BCJ-0687R -CD73-manuscript-revised-Supplementary-file-2-volcano-plot.pdf

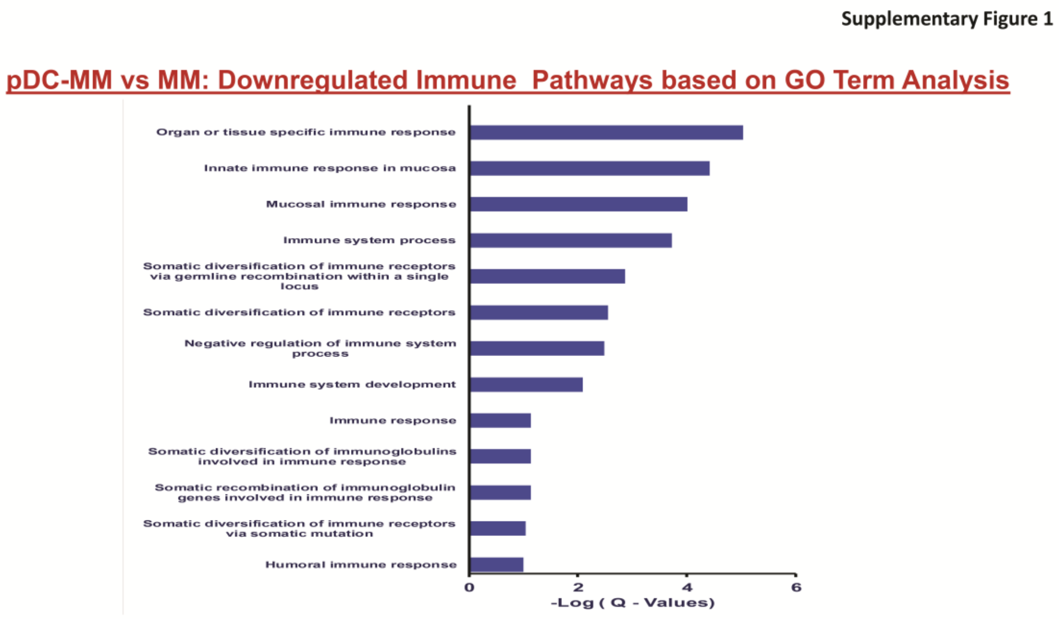

Supplementary Figure 1

Supplementary Figure 2A

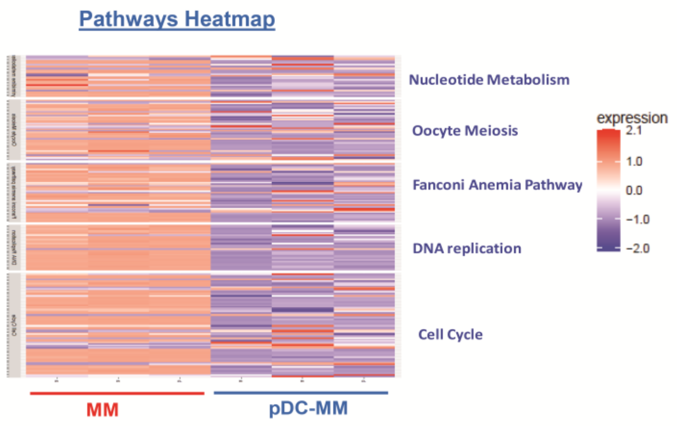

Supplementary Figure 2B

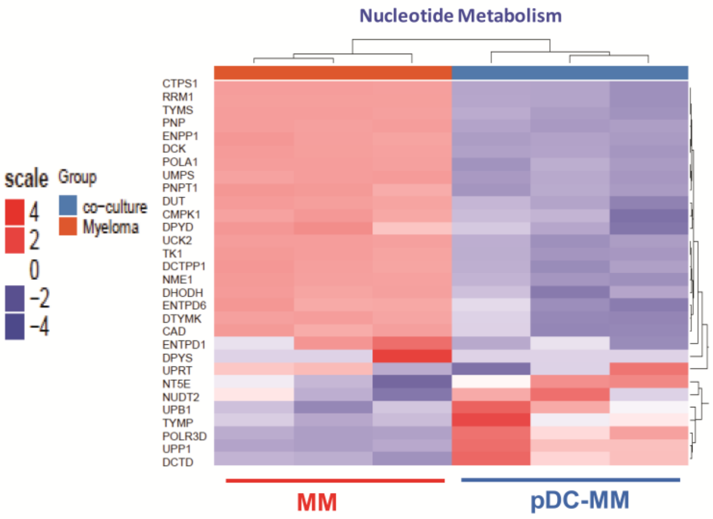

Supplementary Figure 2

Supplementary Figure 3

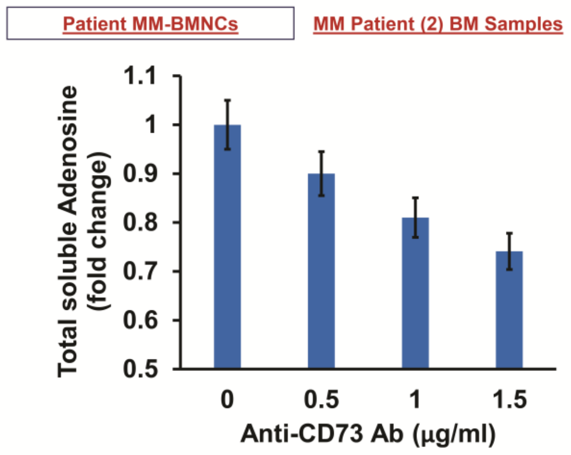

Supplementary Figure 3

Supplementary Figure 4

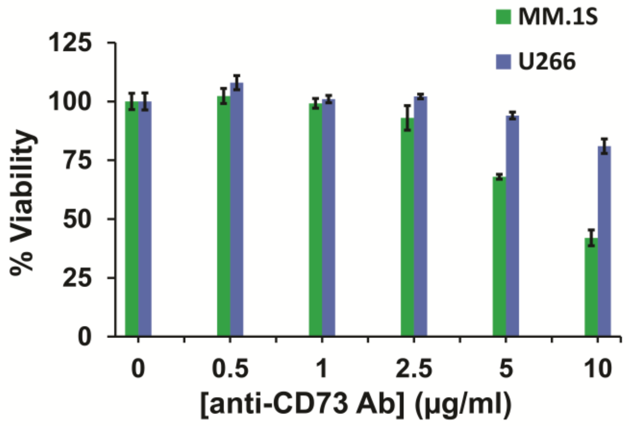

Supplementary Figure 4

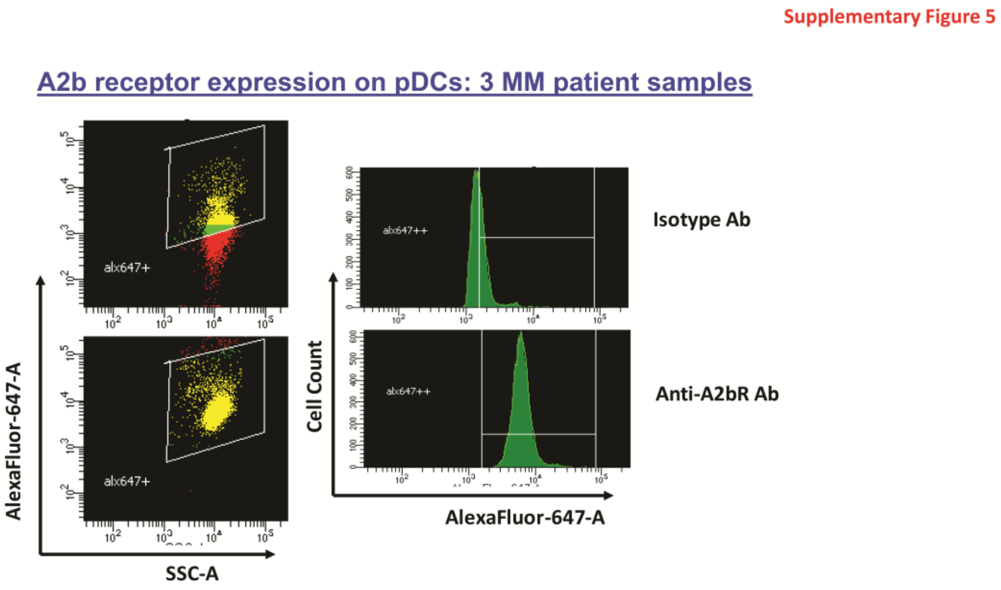

Supplementary Figure 5

Supplementary Figure 6

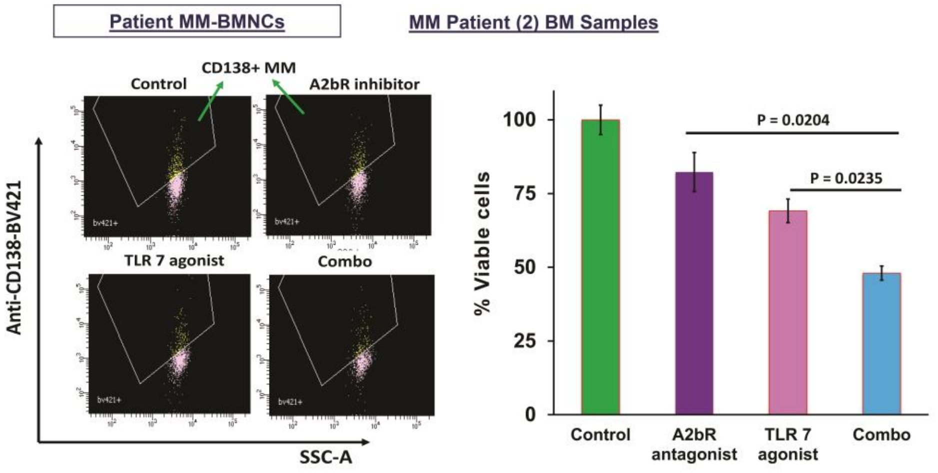

Supplementary Figure 6
