# Supplementary figures and images for: Identification and validation of ecto-5' nucleotidase as an immunotherapeutic target in multiple myeloma
Source: Blood Cancer J. 2022 Apr 1;12(4):50. doi: 10.1038/s41408-022-00635-3 (PMC8976016; doi:10.1038/s41408-022-00635-3)

# Myeloma\_v\_coculture

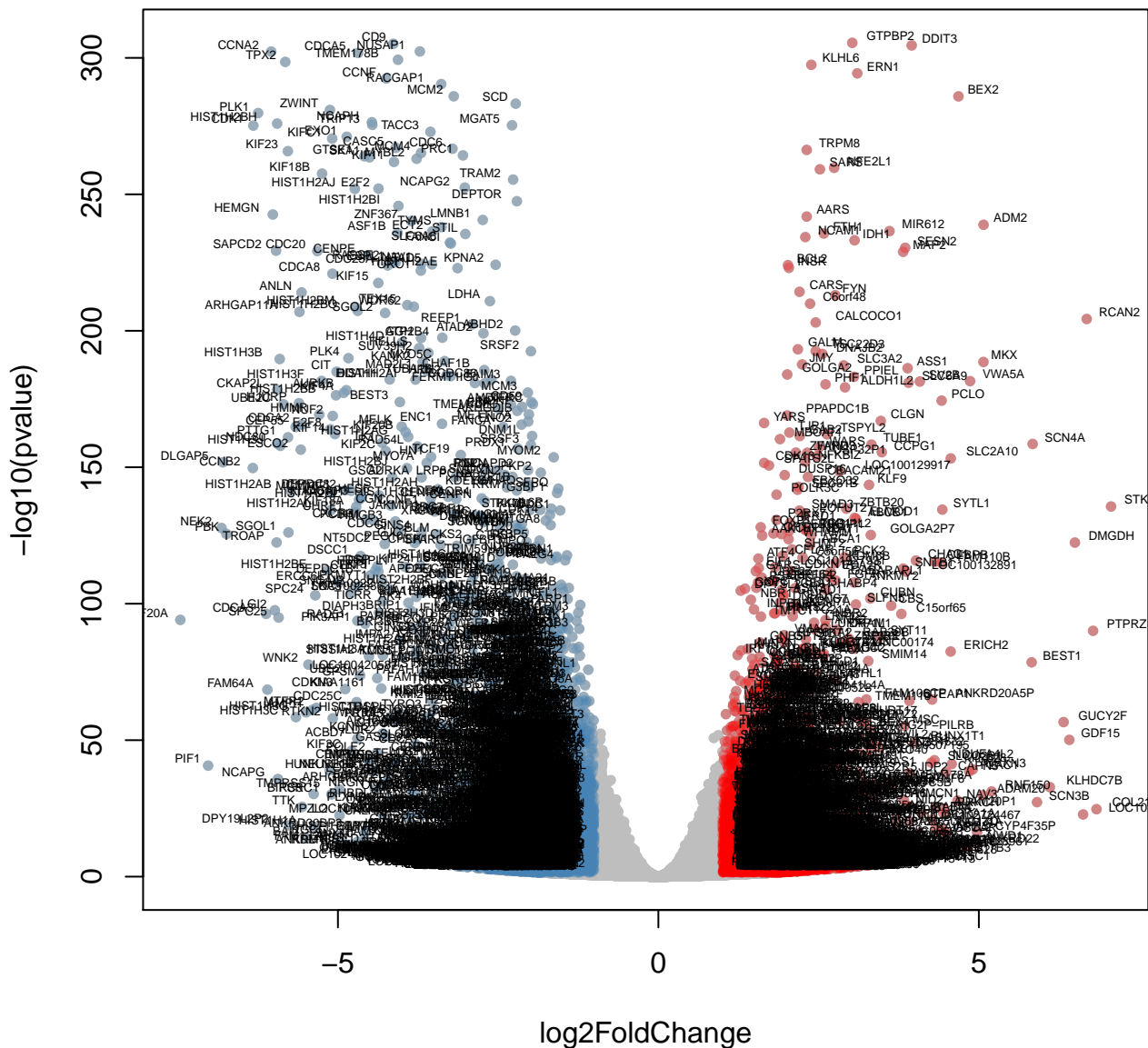

Supplement: Supplementary file 3 — Supplementary-file-2-volcano-plot.pdf [file 41408_2022_635_MOESM3_ESM.pdf]
