# Supplementary material for: Identification and validation of ecto-5' nucleotidase as an immunotherapeutic target in multiple myeloma
Source: Blood Cancer J. 2022 Apr 1;12(4):50. doi: 10.1038/s41408-022-00635-3 (PMC8976016; doi:10.1038/s41408-022-00635-3)
Supplement: Supplementary file 4 — Authors-agreement-on-Change-of-authorship [file 41408_2022_635_MOESM4_ESM.pdf]

Date: 12/20/21

Dr. S. Vincent Rajkumar, MD
Dr. Ayalew Tefferi, MD.
Editors-in-Chief, Blood Cancer Journal
Blood Cancer Journal Editorial Office, Nature Publishing Group
Springer Nature, The Macmillan Building
4 Crinan Street London, UK
Email: bcj@nature.com

Re: Include Dr. Yu-Tzu Tai as coauthor of the revised manuscript 21-BCJ-0687R

Dear Drs. Rajkumar and Tefferi,

All the coauthors from the earlier version of the manuscript, including myself, have agreed to include Dr. Yu-Tzu Tai as one the authors of the revised manuscript. All the email responses have been included in this file.

Thank you,

Arghya Ray
arghya_ray@dfci.harvard.edu

- - - - - - - - - - - - - -

Re: BCJ/CD73

Yan Song

Gmail <song.249@gmail.com>

Thu 12/16/2021 11:13 PM

To:

- Ray, Arghya <Arghya_Ray@dfci.harvard.edu>

Cc:

- Anderson, Kenneth Carl,M.D. <Kenneth_Anderson@dfci.harvard.edu>;
- Chauhan, Dharminder <Dharminder_Chauhan@dfci.harvard.edu>;
- Du, Ting <Ting_Du@DFCI.HARVARD.EDU>;
- Buon, Leutz <leutz_buon@dfci.harvard.edu>

External Email - Use Caution

I agree.

Thank you all.

On Dec 16, 2021, at 16:56, Ray, Arghya <Arghya_Ray@dfci.harvard.edu> wrote:

Hello Everyone,

As a part of (our CD73) manuscript production process, the production editor of the Blood Cancer Journal has requested that all the authors agree upon the addition of Dr. Yu-Tzu Tai as one the authors in the revised manuscript.
Y-YT provided the patient samples, and we have included her name as one of the authors in our revised manuscript.

Identification and Validation of Ecto-5' Nucleotidase as an Immunotherapeutic Target in Multiple Myeloma

Arghya Ray, Ph.D, Yan Song, PhD, Ting Du, Ph.D, Leutz Buon, Yu-Tzu Tai, PhD, Dharminder Chauhan*¶, Ph.D and Kenneth C Anderson*¶, M.D.

The manuscript has already been accepted, but we still need to fulfil the pre-production formalities.

Kindly reply to this email confirming that all of you agree to these changes.
Once I collect all these replies, I'll combine all the co-authors' email responses in one document and upload the file to BCJ.

Thank you all,

Arghya
- - - - - - - - - - - - -

Re: BCJ/CD73
Du, Ting <Ting_Du@DFCI.HARVARD.EDU>

Thu 12/16/2021 5:03 PM
To:

- Ray, Arghya <Arghya_Ray@dfci.harvard.edu>

Hi Arghya,
I agree to all these changes including the addition of Dr. Yu-Tzu Tai as one the authors of this paper.
Thanks,

TING DU

Dana-Farber Cancer Institute
Harvard Medical School
Mayer 553
450 Brookline Ave
Boston MA 02115

From: "Ray, Arghya" <Arghya_Ray@dfci.harvard.edu>
Date: Thursday, December 16, 2021 at 4:56 PM
To: "Anderson, Kenneth Carl,M.D." <Kenneth_Anderson@dfci.harvard.edu>, "Chauhan, Dharminder" <Dharminder_Chauhan@dfci.harvard.edu>, "Du, Ting" <Ting_Du@DFCI.HARVARD.EDU>, Yan Song <song.249@gmail.com>, "Du, Ting" <Ting_Du@DFCI.HARVARD.EDU>, "Buon, Leutz" <leutz_buon@dfci.harvard.edu>
Subject: BCJ/CD73

Hello Everyone,

As a part of (our CD73) manuscript production process, the production editor of the Blood Cancer Journal has requested that all the authors agree upon the addition of Dr. Yu-Tzu Tai as one the authors in the revised manuscript.
Y-YT provided the patient samples, and we have included her name as one of the authors in our revised manuscript.

Identification and Validation of Ecto-5' Nucleotidase as an Immunotherapeutic Target in Multiple Myeloma

Arghya Ray, Ph.D, Yan Song, PhD, Ting Du, Ph.D, Leutz Buon, Yu-Tzu Tai, PhD, Dharminder Chauhan*¶, Ph.D and Kenneth C Anderson*¶, M.D.

The manuscript has already been accepted, but we still need to fulfil the pre-production formalities.

Kindly reply to this email confirming that all of you agree to these changes.
Once I collect all these replies, I'll combine all the co-authors' email responses in one document and upload the file to BCJ.

Thank you all,

Arghya

- - - - - - - - - - - - -

RE: BCJ/CD73

Buon, Leutz <leutz_buon@dfci.harvard.edu>
Fri 12/17/2021 7:50 PM
To:

- Ray, Arghya <Arghya_Ray@dfci.harvard.edu>;
- Anderson, Kenneth Carl,M.D. <Kenneth_Anderson@dfci.harvard.edu>;
- Chauhan, Dharminder <Dharminder_Chauhan@dfci.harvard.edu>;
- Du, Ting <Ting_Du@DFCI.HARVARD.EDU>;
- Yan Song <song.249@gmail.com>;
- Du, Ting <Ting_Du@DFCI.HARVARD.EDU>

I agree.
Thanks,
Leutz.

From: Ray, Arghya <Arghya_Ray@dfci.harvard.edu>
Sent: Thursday, December 16, 2021 4:57 PM
To: Anderson, Kenneth Carl,M.D. <Kenneth_Anderson@dfci.harvard.edu>; Chauhan, Dharminder <Dharminder_Chauhan@dfci.harvard.edu>; Du, Ting <Ting_Du@DFCI.HARVARD.EDU>; Yan Song <song.249@gmail.com>; Du, Ting <Ting_Du@DFCI.HARVARD.EDU>; Buon, Leutz <leutz_buon@dfci.harvard.edu>
Subject: BCJ/CD73

Hello Everyone,

As a part of (our CD73) manuscript production process, the production editor of the Blood Cancer Journal has requested that all the authors agree upon the addition of Dr. Yu-Tzu Tai as one the authors in the revised manuscript.
Y-YT provided the patient samples, and we have included her name as one of the authors in our revised manuscript.

Identification and Validation of Ecto-5' Nucleotidase as an Immunotherapeutic Target in Multiple Myeloma

Arghya Ray, Ph.D, Yan Song, PhD, Ting Du, Ph.D, Leutz Buon, Yu-Tzu Tai, PhD, Dharminder Chauhan*¶, Ph.D and Kenneth C Anderson*¶, M.D.

The manuscript has already been accepted, but we still need to fulfil the pre-production formalities.

Kindly reply to this email confirming that all of you agree to these changes.
Once I collect all these replies, I'll combine all the co-authors' email responses in one document and upload the file to BCJ.

Thank you all,

Arghya

- - - - - - - - - -

Re: BCJ/CD73
Chauhan, Dharminder <Dharminder_Chauhan@dfci.harvard.edu>
Thu 12/16/2021 5:49 PM
To:

- Ray, Arghya <Arghya_Ray@dfci.harvard.edu>

Cc:

- Anderson, Kenneth Carl,M.D. <Kenneth_Anderson@dfci.harvard.edu>;
- Du, Ting <Ting_Du@DFCI.HARVARD.EDU>;
- Yan Song <song.249@gmail.com>;
- Buon, Leutz <leutz_buon@dfci.harvard.edu>

Sure thx

On Dec 16, 2021, at 4:56 PM, Ray, Arghya <Arghya_Ray@dfci.harvard.edu> wrote:

Hello Everyone,

As a part of (our CD73) manuscript production process, the production editor of the Blood Cancer Journal has requested that all the authors agree upon the addition of Dr. Yu-Tzu Tai as one the authors in the revised manuscript.
Y-YT provided the patient samples, and we have included her name as one of the authors in our revised manuscript.

Identification and Validation of Ecto-5' Nucleotidase as an Immunotherapeutic Target in Multiple Myeloma

Arghya Ray, Ph.D, Yan Song, PhD, Ting Du, Ph.D, Leutz Buon, Yu-Tzu Tai, PhD, Dharminder Chauhan*¶, Ph.D and Kenneth C Anderson*¶, M.D.

The manuscript has already been accepted, but we still need to fulfil the pre-production formalities.

Kindly reply to this email confirming that all of you agree to these changes.
Once I collect all these replies, I'll combine all the co-authors' email responses in one document and upload the file to BCJ.

Thank you all,

Arghya

- - - - - - - -

Re: BCJ/CD73

Anderson, Kenneth Carl,M.D. <Kenneth_Anderson@dfci.harvard.edu>
Thu 12/16/2021 5:07 PM
To:

- Ray, Arghya <Arghya_Ray@dfci.harvard.edu>;
- Chauhan, Dharminder <Dharminder_Chauhan@dfci.harvard.edu>;
- Du, Ting <Ting_Du@DFCI.HARVARD.EDU>;
- Yan Song <song.249@gmail.com>;
- Buon, Leutz <leutz_buon@dfci.harvard.edu>

I agree

From: "Ray, Arghya" <Arghya_Ray@dfci.harvard.edu>
Date: Thursday, December 16, 2021 at 4:56 PM
To: kenneth anderson <Kenneth_Anderson@dfci.harvard.edu>, Dharminder Chauhan <Dharminder_Chauhan@dfci.harvard.edu>, "Du, Ting"

<Ting_Du@DFCI.HARVARD.EDU>, Yan Song <song.249@gmail.com>, "Du, Ting" <Ting_Du@DFCI.HARVARD.EDU>, "Buon, Leutz" <leutz_buon@dfci.harvard.edu>
Subject: BCJ/CD73

Hello Everyone,

As a part of (our CD73) manuscript production process, the production editor of the Blood Cancer Journal has requested that all the authors agree upon the addition of Dr. Yu-Tzu Tai as one the authors in the revised manuscript.
Y-YT provided the patient samples, and we have included her name as one of the authors in our revised manuscript.

Identification and Validation of Ecto-5' Nucleotidase as an Immunotherapeutic Target in Multiple Myeloma

Arghya Ray, Ph.D, Yan Song, PhD, Ting Du, Ph.D, Leutz Buon, Yu-Tzu Tai, PhD, Dharminder Chauhan*¶, Ph.D and Kenneth C Anderson*¶, M.D.

The manuscript has already been accepted, but we still need to fulfil the pre-production formalities.

Kindly reply to this email confirming that all of you agree to these changes.
Once I collect all these replies, I'll combine all the co-authors' email responses in one document and upload the file to BCJ.

Thank you all,

Arghya
