# Supplementary material for: Identification and validation of ecto-5' nucleotidase as an immunotherapeutic target in multiple myeloma
Source: Blood Cancer J. 2022 Apr 1;12(4):50. doi: 10.1038/s41408-022-00635-3 (PMC8976016; doi:10.1038/s41408-022-00635-3)
Supplement: Supplementary file 5 — Authors-agreement-on-Change-of-authorship: Doc file [file 41408_2022_635_MOESM5_ESM.docx]

Date: 12/20/21

Dr. S. Vincent Rajkumar, MD

Dr. Ayalew Tefferi, MD.

Editors-in-Chief, Blood Cancer Journal

Blood Cancer Journal Editorial Office, Nature Publishing Group

Springer Nature, The Macmillan Building

4 Crinan Street London, UK

Email: [bcj@nature.com](mailto:bcj@nature.com)

Re: Include Dr. Yu-Tzu Tai as coauthor of the revised manuscript 21-BCJ-0687R

Dear Drs. Rajkumar and Tefferi,

All the coauthors from the earlier version of the manuscript, including myself, have agreed to include Dr. Yu-Tzu Tai as one the authors of the revised manuscript. All the email responses have been included in this file.

Thank you,

Arghya Ray

arghya_ray@dfci.harvard.edu

**- - - - - - - - - - - - -**

**Re: BCJ/CD73**

**Yan Song**

**Gmail <song.249@gmail.com>**

Thu 12/16/2021 11:13 PM

To:

- Ray, Arghya <Arghya_Ray@dfci.harvard.edu>

Cc:

- Anderson, Kenneth Carl,M.D. <Kenneth_Anderson@dfci.harvard.edu>;
- Chauhan, Dharminder <Dharminder_Chauhan@dfci.harvard.edu>;
- Du, Ting <Ting_Du@DFCI.HARVARD.EDU>;
- Buon, Leutz <leutz_buon@dfci.harvard.edu>

**External Email - Use Caution**

I agree.

Thank you all.

On Dec 16, 2021, at 16:56, Ray, Arghya <Arghya_Ray@dfci.harvard.edu> wrote:

﻿

Hello Everyone,

As a part of (our CD73) manuscript production process, the production editor of the Blood Cancer Journal has requested that all the authors agree upon the addition of Dr. Yu-Tzu Tai as one the authors in the revised manuscript.

Y-YT provided the patient samples, and we have included her name as one of the authors in our revised manuscript.

**Identification and Validation of Ecto-5’ Nucleotidase as an Immunotherapeutic Target in Multiple Myeloma**

Arghya Ray, Ph.D, Yan Song, PhD, Ting Du, Ph.D, Leutz Buon, Yu-Tzu Tai, PhD, Dharminder Chauhan*¶, Ph.D and Kenneth C Anderson*¶, M.D.

The manuscript has already been accepted, but we still need to fulfil the pre-production  formalities.

Kindly reply to this email   confirming that all of you agree to these changes.

Once I collect all these replies, I'll combine all the co-authors’ email responses in one document and upload the file to BCJ.

Thank you all,

Arghya

- - - - - - - - - - - - -

**Re: BCJ/CD73**

**Du, Ting <Ting_Du@DFCI.HARVARD.EDU**>

Thu 12/16/2021 5:03 PM

To:

- Ray, Arghya <Arghya_Ray@dfci.harvard.edu>

Hi Arghya,

I agree to all these changes including the addition of  Dr. Yu-Tzu Tai as one the authors of this paper.

Thanks,

TING DU

Dana-Farber Cancer Institute

Harvard Medical School

Mayer 553

450 Brookline Ave

Boston MA 02115

**From:**"Ray, Arghya" <Arghya_Ray@dfci.harvard.edu>
**Date:**Thursday, December 16, 2021 at 4:56 PM
**To:**"Anderson, Kenneth Carl,M.D." <Kenneth_Anderson@dfci.harvard.edu>, "Chauhan, Dharminder" <Dharminder_Chauhan@dfci.harvard.edu>, "Du, Ting" <Ting_Du@DFCI.HARVARD.EDU>, Yan Song <song.249@gmail.com>, "Du, Ting" <Ting_Du@DFCI.HARVARD.EDU>, "Buon, Leutz" <leutz_buon@dfci.harvard.edu>
**Subject:**BCJ/CD73

Hello Everyone,

As a part of (our CD73) manuscript production process, the production editor of the Blood Cancer Journal has requested that all the authors agree upon the addition of Dr. Yu-Tzu Tai as one the authors in the revised manuscript.

Y-YT provided the patient samples, and we have included her name as one of the authors in our revised manuscript.

**Identification and Validation of Ecto-5’ Nucleotidase as an Immunotherapeutic Target in Multiple Myeloma**

Arghya Ray, Ph.D, Yan Song, PhD, Ting Du, Ph.D, Leutz Buon, Yu-Tzu Tai, PhD, Dharminder Chauhan*¶, Ph.D and Kenneth C Anderson*¶, M.D.

The manuscript has already been accepted, but we still need to fulfil the pre-production  formalities.

Kindly reply to this email   confirming that all of you agree to these changes.

Once I collect all these replies, I'll combine all the co-authors’ email responses in one document and upload the file to BCJ.

Thank you all,

Arghya

- - - - - - - - - - - - -

**RE: BCJ/CD73**

**Buon, Leutz <leutz_buon@dfci.harvard.edu>**

Fri 12/17/2021 7:50 PM

To:

- Ray, Arghya <Arghya_Ray@dfci.harvard.edu>;
- Anderson, Kenneth Carl,M.D. <Kenneth_Anderson@dfci.harvard.edu>;
- Chauhan, Dharminder <Dharminder_Chauhan@dfci.harvard.edu>;
- Du, Ting <Ting_Du@DFCI.HARVARD.EDU>;
- Yan Song <song.249@gmail.com>;
- Du, Ting <Ting_Du@DFCI.HARVARD.EDU>

I agree.

Thanks,

Leutz.

**From:** Ray, Arghya <Arghya_Ray@dfci.harvard.edu>
**Sent:** Thursday, December 16, 2021 4:57 PM
**To:** Anderson, Kenneth Carl,M.D. <Kenneth_Anderson@dfci.harvard.edu>; Chauhan, Dharminder <Dharminder_Chauhan@dfci.harvard.edu>; Du, Ting <Ting_Du@DFCI.HARVARD.EDU>; Yan Song <song.249@gmail.com>; Du, Ting <Ting_Du@DFCI.HARVARD.EDU>; Buon, Leutz <leutz_buon@dfci.harvard.edu>
**Subject:** BCJ/CD73

Hello Everyone,

As a part of (our CD73) manuscript production process, the production editor of the Blood Cancer Journal has requested that all the authors agree upon the addition of Dr. Yu-Tzu Tai as one the authors in the revised manuscript.

Y-YT provided the patient samples, and we have included her name as one of the authors in our revised manuscript.

**Identification and Validation of Ecto-5’ Nucleotidase as an Immunotherapeutic Target in Multiple Myeloma**

Arghya Ray, Ph.D, Yan Song, PhD, Ting Du, Ph.D, Leutz Buon, Yu-Tzu Tai, PhD, Dharminder Chauhan*¶, Ph.D and Kenneth C Anderson*¶, M.D.

The manuscript has already been accepted, but we still need to fulfil the pre-production  formalities.

Kindly reply to this email   confirming that all of you agree to these changes.

Once I collect all these replies, I'll combine all the co-authors’ email responses in one document and upload the file to BCJ.

Thank you all,

Arghya

- - - - - - - - - -

**Re: BCJ/CD73**

**Chauhan, Dharminder <Dharminder_Chauhan@dfci.harvard.edu>**

Thu 12/16/2021 5:49 PM

To:

- Ray, Arghya <Arghya_Ray@dfci.harvard.edu>

Cc:

- Anderson, Kenneth Carl,M.D. <Kenneth_Anderson@dfci.harvard.edu>;
- Du, Ting <Ting_Du@DFCI.HARVARD.EDU>;
- Yan Song <song.249@gmail.com>;
- Buon, Leutz <leutz_buon@dfci.harvard.edu>

Sure thx

On Dec 16, 2021, at 4:56 PM, Ray, Arghya <Arghya_Ray@dfci.harvard.edu> wrote:

﻿

Hello Everyone,

As a part of (our CD73) manuscript production process, the production editor of the Blood Cancer Journal has requested that all the authors agree upon the addition of Dr. Yu-Tzu Tai as one the authors in the revised manuscript.

Y-YT provided the patient samples, and we have included her name as one of the authors in our revised manuscript.

**Identification and Validation of Ecto-5’ Nucleotidase as an Immunotherapeutic Target in Multiple Myeloma**

Arghya Ray, Ph.D, Yan Song, PhD, Ting Du, Ph.D, Leutz Buon, Yu-Tzu Tai, PhD, Dharminder Chauhan*¶, Ph.D and Kenneth C Anderson*¶, M.D.

The manuscript has already been accepted, but we still need to fulfil the pre-production  formalities.

Kindly reply to this email   confirming that all of you agree to these changes.

Once I collect all these replies, I'll combine all the co-authors’ email responses in one document and upload the file to BCJ.

Thank you all,

Arghya

- - - - - - - -

**Re: BCJ/CD73**

**Anderson, Kenneth Carl,M.D. <Kenneth_Anderson@dfci.harvard.edu>**

Thu 12/16/2021 5:07 PM

To:

- Ray, Arghya <Arghya_Ray@dfci.harvard.edu>;
- Chauhan, Dharminder <Dharminder_Chauhan@dfci.harvard.edu>;
- Du, Ting <Ting_Du@DFCI.HARVARD.EDU>;
- Yan Song <song.249@gmail.com>;
- Buon, Leutz <leutz_buon@dfci.harvard.edu>

I agree

**From:**"Ray, Arghya" <Arghya_Ray@dfci.harvard.edu>
**Date:**Thursday, December 16, 2021 at 4:56 PM
**To:**kenneth anderson <Kenneth_Anderson@dfci.harvard.edu>, Dharminder Chauhan <Dharminder_Chauhan@dfci.harvard.edu>, "Du, Ting" <Ting_Du@DFCI.HARVARD.EDU>, Yan Song <song.249@gmail.com>, "Du, Ting" <Ting_Du@DFCI.HARVARD.EDU>, "Buon, Leutz" <leutz_buon@dfci.harvard.edu>
**Subject:**BCJ/CD73

Hello Everyone,

As a part of (our CD73) manuscript production process, the production editor of the Blood Cancer Journal has requested that all the authors agree upon the addition of Dr. Yu-Tzu Tai as one the authors in the revised manuscript.

Y-YT provided the patient samples, and we have included her name as one of the authors in our revised manuscript.

**Identification and Validation of Ecto-5’ Nucleotidase as an Immunotherapeutic Target in Multiple Myeloma**

Arghya Ray, Ph.D, Yan Song, PhD, Ting Du, Ph.D, Leutz Buon, Yu-Tzu Tai, PhD, Dharminder Chauhan*¶, Ph.D and Kenneth C Anderson*¶, M.D.

The manuscript has already been accepted, but we still need to fulfil the pre-production  formalities.

Kindly reply to this email   confirming that all of you agree to these changes.

Once I collect all these replies, I'll combine all the co-authors’ email responses in one document and upload the file to BCJ.

Thank you all,

Arghya
